# Seasonal vaccination against malaria: a potential use for an imperfect malaria vaccine

**DOI:** 10.1186/s12936-017-1841-9

**Published:** 2017-05-02

**Authors:** Brian Greenwood, Alassane Dicko, Issaka Sagara, Issaka Zongo, Halidou Tinto, Matthew Cairns, Irene Kuepfer, Paul Milligan, Jean-Bosco Ouedraogo, Ogobara Doumbo, Daniel Chandramohan

**Affiliations:** 1 Faculty of Infectious and Tropical Diseases, London School of Hygiene and Tropical Medicine, Keppel St., London, WC1E 7HT UK; 2 Malaria Research and Training Centre, University of Sciences Techniques and Technologies, Bamako, Mali; 3 Institut de Recherche en Sciences de la Santé, Bobo-Dioulasso, Burkina Faso; 4 Faculty of Epidemiology and Public Health, London School of Hygiene and Tropical Medicine, London, UK

**Keywords:** Seasonal malaria transmission, Seasonal malaria chemoprevention, Seasonal malaria vaccination

## Abstract

In many parts of the African Sahel and sub-Sahel, where malaria remains a major cause of mortality and morbidity, transmission of the infection is highly seasonal. Seasonal malaria chemoprevention (SMC), which involves administration of a full course of malaria treatment to young children at monthly intervals during the high transmission season, is proving to be an effective malaria control measure in these areas. However, SMC does not provide complete protection and it is demanding to deliver for both families and healthcare givers. Furthermore, there is a risk of the emergence in the future of resistance to the drugs, sulfadoxine–pyrimethamine and amodiaquine, that are currently being used for SMC. Substantial progress has been made in the development of malaria vaccines during the past decade and one malaria vaccine, RTS,S/AS01, has received a positive opinion from the European Medicines Authority and will soon be deployed in large-scale, pilot implementation projects in sub-Saharan Africa. A characteristic feature of this vaccine, and potentially of some of the other malaria vaccines under development, is that they provide a high level of efficacy during the period immediately after vaccination, but that this wanes rapidly, perhaps because it is difficult to develop effective immunological memory to malaria antigens in subjects exposed previously to malaria infection. A potentially effective way of using malaria vaccines with high initial efficacy but which provide only a short period of protection could be annual, mass vaccination campaigns shortly before each malaria transmission season in areas where malaria transmission is confined largely to a few months of the year.

## The seasonality of malaria

**Fig. 1:**
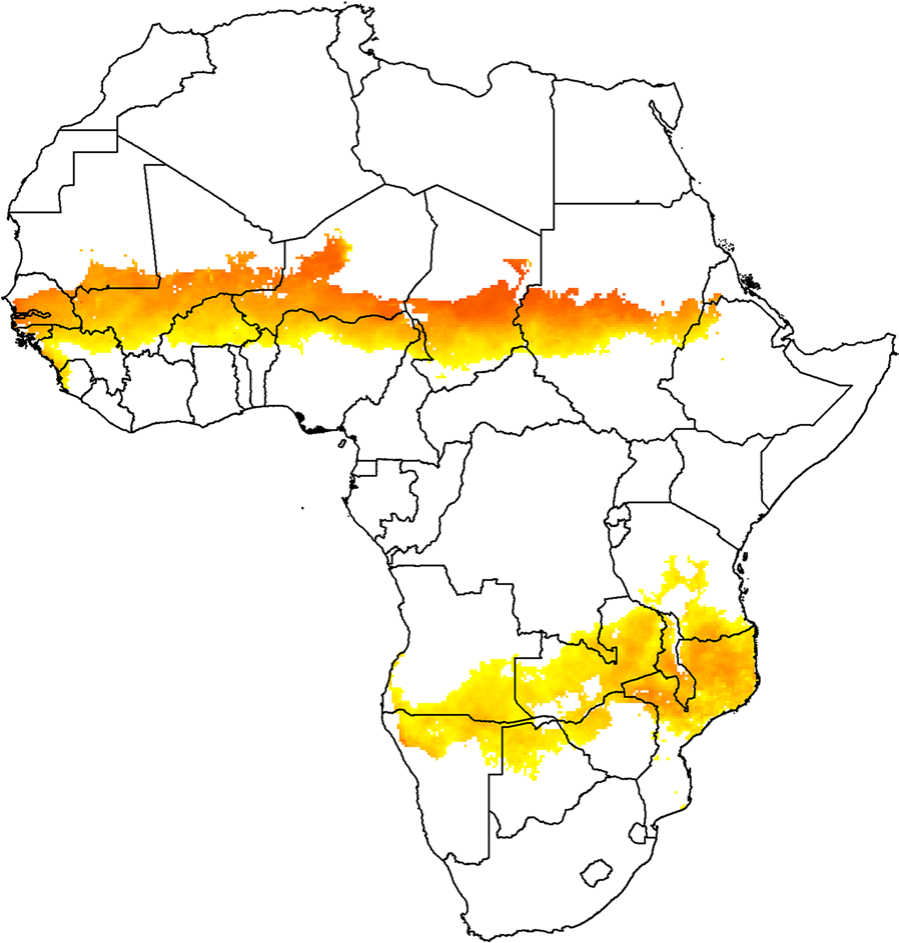
A map of sub-Saharan Africa showing the areas where malaria transmission is likely to be highly seasonal. *Orange areas* indicate where more than 60% of annual rainfall occurs within 3 months of the year, and where malaria incidence is estimated to exceed 100 cases per 1000 children per year (adapted from Cairns et al. [1])

Malaria infection shows some degree of seasonality in nearly all areas where the infection is endemic. In extreme cases, for example areas bordering a desert, transmission may be limited to only a few weeks in a year and occur at irregular intervals. In large parts of Sahelian and sub-Sahelian Africa, most transmission occurs during just a few months of the year, although there have been few observational studies which have recorded in detail the incidence of malaria by month of year over a period of several years. In tropical Africa, where the temperature is suitable for malaria transmission for most of the year, the seasonality of malaria transmission is determined largely by seasonal changes in rainfall. By comparing the incidence of malaria by month of year with the monthly rainfall in areas where both have been determined, it has been possible to estimate from rainfall patterns, the areas of Africa where malaria is likely to be concentrated during 3 or 4 months of the year [1]. These lie predominantly in the Sahelian and sub-Sahelian regions of Africa (Fig. 1), where the burden of malaria continues to be very high.

## Seasonal malaria chemoprevention

On the assumption that in areas with highly seasonal malaria, it would be necessary to provide protection against malaria for only a few months of the year to obtain highly effective control of clinical malaria, a series of trials were conducted in countries of the Sahel and sub-Sahel in the 2000s which evaluated the impact of providing young children with a treatment course of an effective anti-malarial combination at monthly intervals for three or four months during the peak period of malaria transmission. This intervention, known initially as intermittent preventive treatment in children (IPTc) and now as seasonal malaria chemoprevention (SMC), proved highly effective when evaluated in clinical trials, reducing the incidence of uncomplicated malaria by 82% (95% CI 75, 87%) and that of severe malaria by 82% (95% CI 48, 94%), and SMC probably reduced childhood deaths [2]. Similar, but slightly lower estimates of the degree of protection provided by SMC were obtained in a Cochrane review of the efficacy of SMC [3]. On the basis of these findings, WHO’s Malaria Policy Advisory Committee (MPAC) made a recommendation in 2012 that SMC with sulfadoxine pyrimethamine (SP) and amodiaquine (AQ), given at monthly intervals for 3 or 4 months, should be introduced into areas of sub-Saharan Africa where malaria transmission is highly seasonal and the parasite sensitive to these drugs [4].

Following the provision of financial support from UNITAID and other international donors, SMC is now being distributed widely across the Sahel and sub-Sahel with a potential annual target of around 20 million children [1]. Preliminary results suggest that coverage with SMC when rolled out on a large scale through a national programme has generally been high [5] although there is evidence that coverage declines with later rounds of drug administration in some countries. The WHO recommendation on SMC related just to children under the age of 5 years, but there is recent evidence that in countries, such as Senegal, where the main burden of malaria is now in older children, SMC given to children under the age of 10 years is also highly effective and has an indirect ‘herd’ effect, reducing the incidence of malaria in older subjects who did not receive SMC [6].

SMC is, so far, proving to be a success when implemented on a large scale but sustaining its effective delivery will require a long-term commitment from the communities where the intervention is being delivered from national malaria control programmes and from donors. Ensuring that the anti-malarials needed for SMC are available at the right time of the year, which may vary from year to year depending upon rainfall patterns, requires careful planning and logistics. In most large-scale programmes, SMC is delivered through door-to-door visits, although in some programmes mothers and children are expected to gather at a fixed point in their community for drug administration. Twelve contacts per year between child and health care worker are required if each dose of anti-malarial is to be given under direct observation, which is rarely practicable and, if this is not done, there is a danger that children will receive only their first dose of treatment and not a full course. An increase in the proportion of malaria parasites carrying markers of resistance to SP at the end of the malaria transmission season was recorded in some of the initial trials of SMC [2, 7] and, although there is currently no evidence that SMC is failing because of the emergence of resistance, this is a potential threat for the future. Currently, there is no drug combination that could be used to replace SP and AQ in the areas where SMC is being delivered except dihydroartemisinin–piperaquine (DHA–PQ) [8], and a decision to use an artemisinin-based combination therapy on a large scale for chemoprevention in Africa would be controversial in view of the threat of the emergence and spread of artemisinin and piperaquine resistance on the continent. Development of an anti-malarial with a long action specifically for use in chemoprevention in Africa would take several years. For these reasons, it is important to consider measures that could be used in addition to SMC to control malaria in areas where the infection is highly seasonal or, potentially, as an alternative.

## Progress in malaria vaccine development

**Fig. 2:**
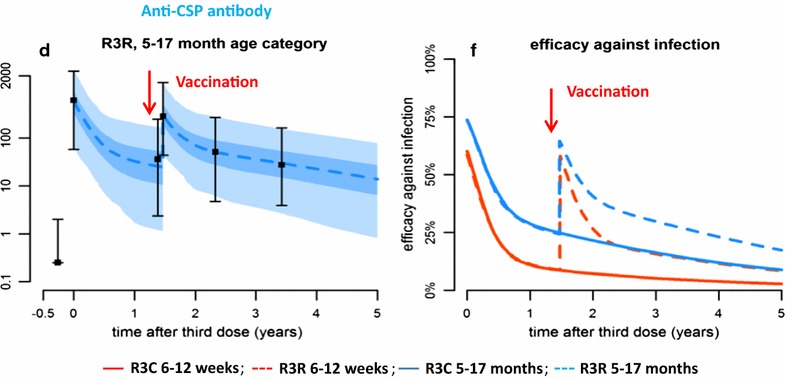
The transient nature of the protection provided by RTS,S/AS01 and the associated changes in anti-CSP antibody concentration following primary vaccination and administration of a booster dose modelled on the basis of the results of phase 2 and phase 3 RTS,S/AS01 efficacy trials. **d** Shows anti-CSP antibody titres and **f** shows the percentage efficacy after primary and booster immunization. *Red lines* indicate infants vaccinated at the age of 6–12 weeks, *blue lines* those vaccinated at the age of 5–17 months. *Dotted lines* indicate children who recived a booster dose and solid lines those who did not (From White et al. [14])

There has been considerable progress in malaria vaccine development during the past decade. Following a large phase 3 trial in seven African countries [9], the pre-erythrocytic vaccine RTS,S/AS01 has received a positive opinion from the European Medicines Agency [10] and will soon be deployed in large-scale, pilot implementation projects. A second vaccine, the irradiated sporozoites vaccine (PfSPZ), is near to pivotal phase 3 trials [11]. Several other pre-erythrocytic and blood stage vaccines have shown efficacy in challenge experiments in volunteers and in endemic populations but, in general, efficacy has only been limited. A characteristic feature of the protection provided by RTS,S/AS01 is its relatively short duration. A high level of protection is found during the first few months after administration in both young children and adults but this wanes rapidly after primary immunization [9, 12, 13]. This loss of protection is accompanied by a similar rapid decline in antibodies to the circumsporozoite antigen (CSP), a partial correlate of protection, despite the fact that the vaccine is given with a powerful adjuvant. Antibody titres and protection can be restored partially by a booster dose of vaccine given 18 months after primary immunization but the enhanced response is again only temporary (Fig. 2) [9, 14]. Little information is available about the duration or protection provided by other malaria vaccines or about the impact of a booster dose. In the case of vaccines based on *Plasmodium falciparum* irradiated sporozoites (PfSPZ), or on live sporozoites given under cover of anti-malarial treatment, it has been shown that some immunized volunteers can sustain protection to challenge with a homologous strain of parasite for 1 to 2 years [15–17]. However, in a recent study conducted in non-immune volunteers, the initial high efficacy of PfSPZ against heterologous challenge was lost 6 months after vaccination [18]. PfSPZ was less efficacious in adult Malian volunteers than in American volunteers [19]. Both pre-erythrocytic and blood stage vaccines have been developed that use two viral vectors given in a prime boost regimen and these have provided some protection in both volunteers and in the field. Viral-vectored vaccines induce very strong T cell response, as measured by ELISPOT, but this T cell response declines rapidly in the months following vaccination [20, 21] suggesting that vaccines based on this delivery system might also provide only a relatively short period of protection.

Why it is proving difficult to induce effective immunological memory and long-term protection with malaria vaccines is uncertain. There is recent evidence that previous exposure to malaria may have an impact on the immunological response to subsequent exposure to malaria antigens [22] and that this might be mediated through generation of atypical B memory cells [23, 24] and/or by depletion of T follicular helper cells (Tfh cells) which play a key role within germinal centres in facilitating the development of immunological memory [25]. Thus, in malaria endemic areas, vaccination of young children prior to exposure to malaria may be a useful approach. However, immune modulation by prior exposure to malaria cannot explain the transitory nature of the protective immune response seen in non-malaria exposed volunteers.

## Optimum use of a vaccine providing only a short period of protection

It is possible that the next generation of malaria vaccines will, like RTS,S/AS01, provide only a relatively short period of high-level protection and imperfect immunological memory. If this proves to be the case, it is important to explore situations in which a vaccine with high initial efficacy but which cannot provide sustained protection could be used most effectively. One such situation is the final stage of an elimination programme, provided that the vaccine protected against infection as well as clinical malaria. In this case the vaccine, given to the whole population, needs to be very safe and effective but does not need to provide sustained protection provided that elimination is achieved. A second use for such a vaccine could be pre-empting or halting an epidemic caused by an unusual set of environmental or social circumstances, such as a civil emergency, which is likely to be only temporary. A third possibility could be use in pregnancy provided the vaccine was shown to be non-teratogenic. Finally, the vaccine could be used as a seasonal vaccine in areas where a high level of transmission is sustained from year to year but is also highly seasonal, with the period of maximum risk matching the duration of protection provided by the vaccine.

## Seasonal malaria vaccination

Influenza provides a paradigm for seasonal vaccination. In many industrialized countries, annual influenza vaccination prior to the influenza season is recommended for children as well as the elderly and immune-compromised [26]. This is necessary because of the need to adjust the vaccine to the prevailing strain of virus but also because of the poor immunological memory induced by most influenza vaccines. A malaria vaccine which provides a high level of initial protection but which is of only short duration could be used in a similar way to influenza vaccines to provide protection to children in areas where the peak period of malaria transmission is limited to a few months each year and where the burden of infection is still high.

Following initial priming of young children, which should be undertaken at as early an age as possible, seasonal vaccination could be used either as a supplement to SMC in areas where the incidence of malaria remains high despite effective delivery of SMC, or as a replacement for SMC if this became necessary due to difficulties in sustaining coverage or compliance or because of the emergence of drug resistance. Even if efficacy declined fairly rapidly after vaccination, it is likely that vaccine would, unlike SMC, provide some protection during the following dry season when some malaria transmission may still occur. Administration of a single dose of vaccine through an annual mass campaign directed at children under the age of 5 or 10 years may be logistically easier than administration of four rounds of treatment each year for both health providers and recipients and could be as cost effective. Coverage with mass vaccination campaigns, for example with the group A meningitis vaccine MenAfriVac^R^, in countries of the Sahel has generally been very high [27].

**Fig. 3:**
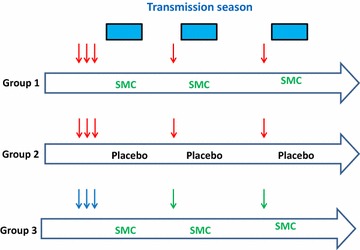
Design of a trial to compare the efficacy of seasonal vaccination with RTS,S/AS01 with SMC with SP + AQ and the impact of the two interventions combined on the incidence of clinical malaria in children under 5 years of age in Burkina Faso and Mali

In order to test this idea, a trial will be conducted in 6000 young children in Burkina Faso and Mali which will investigate whether priming of young children with RTS,S/AS01 followed by a single booster dose at the beginning of the two following malaria transmission seasons is non-inferior to SMC with SP and AQ during the malaria transmission season, or superior over a whole year, and as acceptable and cost effective, and whether combination of the two interventions (SMC and RTS,S/AS01) is superior to either used alone (Fig. 3). The primary trial end-point will be the incidence of clinical malaria detected by passive case detection and there will be several secondary end-points. The trial is powered to have more than 80% power to exclude a difference of 15% in the incidence of clinical malaria over the study period between groups. The trial is set to start in 2017 and last three years.
